# Network-assisted investigation of virulence and antibiotic-resistance systems in *Pseudomonas aeruginosa*

**DOI:** 10.1038/srep26223

**Published:** 2016-05-19

**Authors:** Sohyun Hwang, Chan Yeong Kim, Sun-Gou Ji, Junhyeok Go, Hanhae Kim, Sunmo Yang, Hye Jin Kim, Ara Cho, Sang Sun Yoon, Insuk Lee

**Affiliations:** 1 Department of Biotechnology, College of Life Science and Biotechnology, Yonsei University, Seoul, 120-749, Korea; 2 Center for Systems and Synthetic Biology, University of Texas at Austin, Austin, TX 78712, USA; 3 Department of Microbiology and Immunology, Brain Korea 21 PLUS Project for Medical Science, Yonsei University College of Medicine, Seoul, 120-749, Korea

## Abstract

*Pseudomonas aeruginosa* is a Gram-negative bacterium of clinical significance. Although the genome of PAO1, a prototype strain of *P. aeruginosa*, has been extensively studied, approximately one-third of the functional genome remains unknown. With the emergence of antibiotic-resistant strains of *P. aeruginosa*, there is an urgent need to develop novel antibiotic and anti-virulence strategies, which may be facilitated by an approach that explores *P. aeruginosa* gene function in systems-level models. Here, we present a genome-wide functional network of *P. aeruginosa* genes, PseudomonasNet, which covers 98% of the coding genome, and a companion web server to generate functional hypotheses using various network-search algorithms. We demonstrate that PseudomonasNet-assisted predictions can effectively identify novel genes involved in virulence and antibiotic resistance. Moreover, an antibiotic-resistance network based on PseudomonasNet reveals that *P. aeruginosa* has common modular genetic organisations that confer increased or decreased resistance to diverse antibiotics, which accounts for the pervasiveness of cross-resistance across multiple drugs. The same network also suggests that *P. aeruginosa* has developed mechanism of trade-off in resistance across drugs by altering genetic interactions. Taken together, these results clearly demonstrate the usefulness of a genome-scale functional network to investigate pathogenic systems in *P. aeruginosa*.

*Pseudomonas aeruginosa* is a Gram-negative bacterium of clinical significance. *P. aeruginosa* is an opportunistic human pathogen that can propagate in the abnormal human airway^1^, burn-damaged skin^2^, and artificially implanted organs^3^,^4^, and can also cause hospital-acquired secondary infections in patients with compromised immune reactivity^5^. *P. aeruginosa* infection is often exacerbated by the formation of biofilm, a mode of bacterial growth associated with antibiotic tolerance^6^.

The treatment of *P. aeruginosa* infection faces major challenges due to the constant emergence of antibiotic-resistant variants. Antibiotic resistance to *P. aeruginosa* increases the rate of disease occurrence and mortality^5^,^7^. In contrast, the number of new FDA-approved antibacterial agents has decreased significantly over the past three decades^8^. Alternative strategies are urgently needed for effective *P. aeruginosa* infection control. Recently, an approach to identify chemical agents that can downregulate *P. aeruginosa* virulence without affecting its viability has been attempted^9^. This approach suggests the potential benefit of an anti-virulence strategy, which is in contrast to the predominant anti-viability strategy that has been applied since the discovery of antibiotics.

PAO1, which is one strain of *P. aeruginosa*, has a genome that contains 6.264 million base pairs and 5,572 open reading frames^10^. The PAO1 genome represents one of the largest bacterial genomes; only a few bacterial species, including *Myxococcus xanthus*^11^, *Gemmata obscuriglobus*^12^, and *Mycobacterium smegmatis*^13^ possess larger genomes. The PAO1 genome contains a large number of genes involved in regulation. A gene annotation analysis has shown that PAO1 can produce as many as 487 proteins that either act as transcription factors or are involved in two-component regulatory systems^10^. The versatile adaptability of PAO1 to a myriad of environmental conditions has been attributed to this feature. PAO1 has 2,025 genes, whose cellular functions remain hypothetical or unknown, based on the function class search provided by the Pseudomonas genome website (www.pseudomonas.com). Thus, further investigation is warranted to define the precise cellular functions of poorly characterised genes. In addition to the lack of functional understanding of individual genes, the genetic organisation of traits of clinical importance, such as virulence and drug resistance, remain largely unknown. Although several genome-wide experiments involving forward or reverse genetic screens have been performed to determine clinically important traits, these experiments often miss genes whose knockouts exhibit subtle phenotypes or affect virulence in a specific host environment only^14^.

A prediction-driven genetics approach can complement high-throughput screens by conducting a more careful examination on relatively small sets of highly probable candidates, which can identify the false negatives in high-throughput assays. Recently, predictive functional gene networks inferred from various genomics data have proven useful in the study of *P. aeruginosa*^15^ as well as other bacterial pathogens such as plant pathogens *Fusarium graminearum*^16^ and *Phytophthora infestans*^17^. The integration of functional associations derived from diverse experimental and computational analyses allows for the construction of highly accurate and comprehensive co-functional networks. The guilt-by-association principle, by which two connected genes in the network are likely to have same function, is then used to facilitate the identification of novel genes for virulence and drug resistance. Although the previous functional networks for bacterial pathogens were computationally validated, demonstrating feasibility of identifying novel genes for pathogenicity and drug resistance with experimental validation was not available. In this study, we present a genome-scale functional network of the *P. aeruginosa* strain PAO1, called PseudomonasNet, that maps 203,118 links among 5,456 genes (~98% of the coding genome), whose predictions were validated by experiments. We demonstrate the feasibility of the network-assisted identification of novel genes for virulence and antibiotic resistance with experimental validation. We also show that an antibiotic-resistance network in PseudomonasNet can account for the prevalence of cross-resistance, in which a gene knockout responds in the same direction to multiple drugs (i.e., responds with either increased or decreased resistance). This network also provides mechanistic insights into the trade-off in resistance to different drugs. To provide a more practical contribution to the research community, we have also developed a web-based platform for network-assisted hypothesis generation, which to the best of our knowledge is the first of its kind for *P. aeruginosa*. All the network-assisted predictions that are demonstrated here can be easily reproduced and applied to many other clinically important traits of *P. aeruginosa* using the companion web server (www.inetbio.org/pseudomonasnet/).

## Results

### Construction of a genome-scale co-functional network of *P. aeruginosa* genes

**Figure 1:**
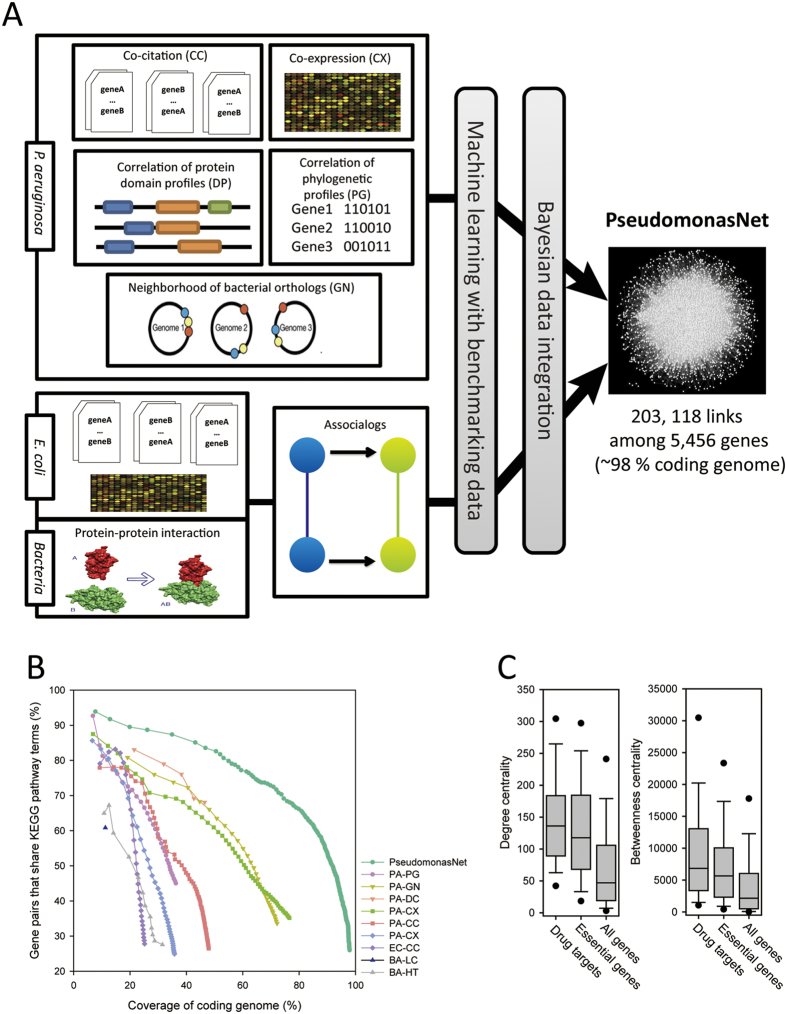
Construction and assessment of PseudomonasNet. (**A**) A summary of the construction of an integrated co-functional network for *P. aeruginosa*. The co-functional links between *P. aeruginosa* genes were derived from nine diverse data sets: five *P. aeruginosa* co-functional networks, including co-citation (CC), co-expression (CX), correlation of protein domain profiles (DP), neighbourhood of bacterial orthologues (GN), and correlation of phylogenetic profiles (PG), and four associalog networks from co-citation and co-expression of *E. coli* orthologues and bacterial protein-protein interactions. PseudomonasNet was constructed based on a machine learning approach with reference gold-standard functional gene pairs that share Gene Ontology biological process annotations using a Bayesian data integration framework. (**B**) The integrated PseudomonasNet and individual component networks were assessed for precision via a comparison to KEGG pathway annotations. We measured the proportion of the gene pairs annotated by KEGG that share same pathway terms for every bin of 1,000 gene pairs from the highest score. The integrated network covers approximately 98% of the *P. aeruginosa* coding genes with superior precision to all individual component networks, which confirms the effectiveness of the data integration in the construction of the genome-scale functional network of the *P. aeruginosa* genes. (**C**) Network centralities of drug targets and essential genes are significantly higher than that of genomic average based on both degree- and betweenness-based scores in PseudomonasNet, which suggests that other genes with high centrality scores are good candidates for drug targets.

The construction of a functional network for *P. aeruginosa* PAO1 is summarised in Fig. 1A and Table 1, and described in detail in the Supplementary Online Methods. Pairs of *P. aeruginosa* genes that operate within the same pathways were inferred from five distinct types of *P. aeruginosa* data: co-citation in Medline articles (PA-CC), co-expression across microarray experiments (PA-CX), correlation of protein domain profiles (PA-DP), correlation of phylogenetic profiles (PA-PG)^18^, and genomic neighbourhoods of bacterial orthologues (PA-GN)^19^. In addition, four sets of orthology-based functional associations (associalogs)^20^ were inferred from the co-citation of *E. coli* genes (EC-CC), co-expression of *E. coli* genes (EC-CX)^21^, bacterial protein-protein interactions derived from high-throughput assays (BA-HT), and literature curation of small-scale analyses (BA-LC). These nine networks were integrated using a Bayesian statistical framework^22^. To benchmark inferred co-functional gene pairs, we used gold-standard *P. aeruginosa* gene pairs that share annotations in the Gene Ontology (GO) biological process database^23^, which included only 906 *P. aeruginosa* genes (~16% of all 5,572 coding genes) with annotations based on reliable evidence (i.e., experimental- or literature-based). The final integrated network, PseudomonasNet, includes 203,118 co-functional links among 5,456 genes, which covers ~98% of the coding genome. Therefore, PseudomonasNet provides new opportunities for functional predictions of many uncharacterised genes.

The pairwise comparisons between the nine component networks showed only small overlaps (Supplementary Fig. S1), which suggests either high complementarity or inaccuracy of the networks. We therefore assessed the overall quality of PseudomonasNet as well as individual component networks using gene pairs that share annotations in the KEGG pathway database^24^, which is independent from the Gene Ontology biological process database used for the network training. All component networks showed reasonably high accuracy for KEGG pathway links, which indicates that the small network overlaps are due to their complementarity rather than inaccuracy. We also observed substantial improvement in both the accuracy and genomic coverage of the integrated PseudomonasNet over individual component networks (Fig. 1B), which indicates that the network has been improved by the integration of various experimental and computational data.

We also examined topological properties of PseudomonasNet. We found all genes except four are connected in the largest component of PseudomonasNet. Distribution of the number of connections indicated that PseudomonasNet is a small-world network with *broad-scale*^25^ (Supplementary Fig. S2), whose connectivity distribution has a power law regime followed by exponential decay of the tail, which is characteristic global topology for task-driven social networks (e.g., Board of directors) or functional protein networks^26^. The broad scale of degree distribution can be attributed to the high network modularity by enrichment of within-group (e.g., within-pathway) connections, implicating that PseudomonasNet retrieves relationships between genes that belong to the same pathways.

### Antimicrobial drug targets are more likely to be hub genes in PseudomonasNet

Bacterial genes that are critical for viability tend to be centralised in the gene or protein network^27^. Such hub microbial genes that have no homologs in the host genome are potential antimicrobial drug targets^28^. To test whether known antibiotic target proteins are more likely to be hubs in PseudomonasNet, we examined the network centrality scores of 73 *P. aeruginosa* orthologues of 93 bacterial proteins that have previously been reported as antimicrobial drug targets^29^. Two different network centrality measures were used for this analysis: degree centrality, in which a gene with more connected neighbours is considered to be more central, and betweenness centrality, in which a gene located on the shortest path between the larger number of gene pairs is more central. We observed significantly higher distribution of network centrality for known antimicrobial drug targets than that for all *P. aeruginosa* genes in PseudomonasNet (Fig. 1C, *P*-value = 2.2e-16 and 4.81e-13 for degree and betweenness centrality, respectively; Wilcoxon rank-sum test), which suggests that PseudomonasNet may be used to predict novel microbial drug targets for the development of antibiotics against *P. aeruginosa.* We found that essential genes^30^ also tend to be hubs in PseudomonasNet and 48 of the 73 drug target (65.8%) are essential genes.

### Algorithms for network-assisted hypothesis generation

The main purpose for the development of PseudomonasNet was to provide experimental biologists with an accessible research platform to generate testable hypothesis about traits of clinical importance. We implemented three complementary network-search algorithms for such hypothesis generation: (i) pathway-centric search, (ii) gene-centric search, and (iii) context-centric search.

**Figure 2:**
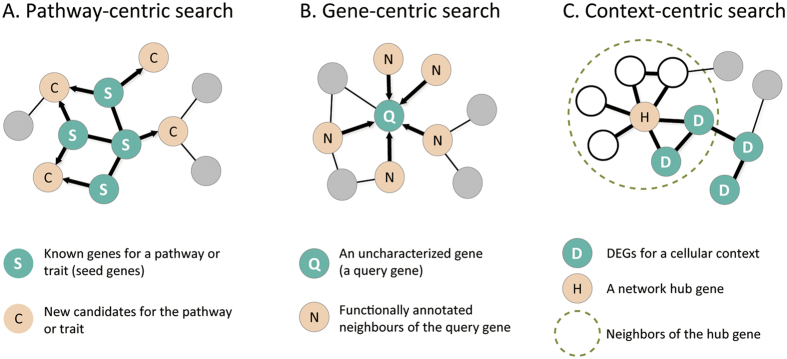
Three network-search algorithms were implemented on the PseudomonasNet web server. (**A**) The pathway-centric search prioritises candidate genes for a pathway or trait by connectivity to the user-provided seed genes. (**B**) The gene-centric search prioritises candidate functional terms (e.g., Gene Ontology biological process terms) for a query gene based on the enrichment of known functional terms among its neighbours. (**C**) The context-centric search prioritises candidate genes for a context (e.g., a clinical condition, such as a drug response) as represented by differential expressed genes (DEGs). The statistical association between the neighbours of a hub gene and the DEGs are measured by a one-tail Fisher’s exact test.

Pathway-centric search (Fig. 2A) starts with a set of known genes for a pathway or trait. Assuming all connected genes in the network are functionally coupled, we expect that known genes for the same pathway or trait are interconnected in PseudomonasNet, and additional genes that are well-connected to the known genes are also likely to be involved in the same pathway or trait. Therefore, if we have known genes for a pathway or a trait of interest, this search method would be the best choice for hypothesis generation. In contrast, gene-centric search (Fig. 2B), which starts with an uncharacterised query gene, can infer the function of the query gene by searching for an enriched function among network neighbours of the query gene.

Context-centric search (Fig. 2C) differs from the two previous algorithms in that it uses differential expressed genes (DEGs) as input. DEGs are a molecular signature of a specific biological context. The key idea of this algorithm is that if the neighbours of a hub gene respond to a certain cellular context, then the hub gene is likely to be involved in the cellular context. If a set of network neighbours of a hub gene has significant overlap with input DEGs for a clinical condition, we may hypothesise that the hub gene is associated with the clinical condition. For given expression profiles of a clinical condition, this network-search method provides an alternative way to identify novel genes involved in pathogenic traits such as antibiotic resistance.

We implemented all three network-search algorithms in the companion web server to PseudomonasNet (www.inetbio.org/pseudomonasnet). For example, the web server reports receiver operating characteristic (ROC) curve which indicates retrieval rate of the user-input genes by PseudomonasNet. ROC analyses for KEGG pathways suggest that PseudomonasNet is highly predictive for many cellular processes (Supplementary Fig. S3). We also examined contribution of *P. aeruginosa* specific data to the pathway prediction by testing a network with no links derived from only other bacterial data (EC-CC, EC-CX, BA-HT, BA-LC of Table 1). We found that a network of Psedomonas-derived links only, which contains 157,395 links (~77.5% of PseudomonasNet) is highly predictive for the same KEGG pathways, but not as much as PsedomonasNet, which suggest that significant portion of predictive power for the *P. aeruginosa* pathways was originated from the links derived from *E. coli* and other bacterial species. Below, we will demonstrate how these network-search algorithms are used to predict novel genes for virulence and antibiotic resistance. All predictions in this manuscript can be reproduced by users with example input data available on the web server.

### PseudomonasNet predicts novel virulence-associated genes

In addition to the computational demonstration of the prediction power of PseudomonasNet as described above, we sought to experimentally validate its usefulness in predicting novel genes associated with *P. aeruginosa* traits related to infection. Although *P. aeruginosa* is a human pathogen, its virulence factors are also effective in exerting cytotoxicity to diverse infection hosts, including mouse^9^, nematode^31^, and plant^32^. In a recent genome-wide screening study using *Caenorhabditis elegans* as an infection host, 41 genes of the *P. aeruginosa* PA14 strain were shown to affect virulence^33^. Orthologues for 38 of these 41 PA14 genes involved in virulence are present in PAO1 (Supplementary Table S1). These orthologues can be used as seed genes to retrieve more virulence-associated genes in PAO1 by PseudomonasNet.

First, we measured the prediction power of PseudomonasNet for the 38 virulence-associated genes. Assuming an accurate functional network with well-connected functionally coherent genes, we expect that virulence genes will score high when the scoring is based on connectivity to known virulence-associated genes. Receiver operating characteristic (ROC) analysis of the ranked virulence-associated genes, which can be summarised by an area under the ROC curve (AUC) score, results in a score of 0.85. This score indicates that PseudomonasNet is highly predictive for virulence in *C. elegans*. Therefore, we may expect that other genes that are highly connected to the 38 virulence genes are also strong candidates.

**Figure 3:**
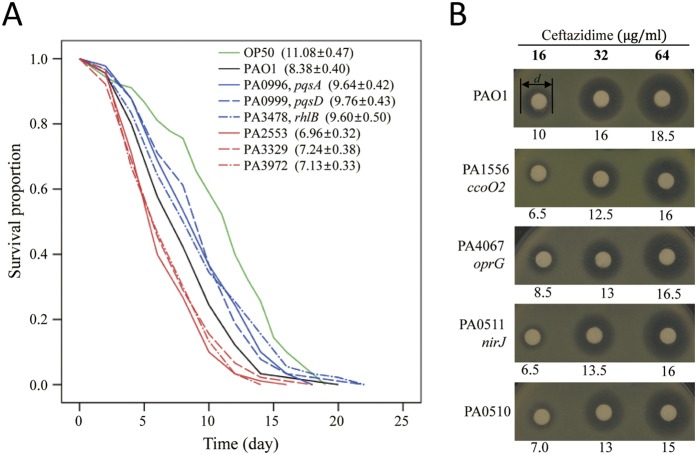
Network-assisted identification of novel genes for virulence and drug resistance. (**A**) The survival curve of *C. elegans* fed with *E. coli* OP50, wild-type and each of six PAO1 mutants. Average survival time in day of 90 worms for each strain is indicated in the parenthesis. Three mutants conferred a significantly increased survival rate (blue lines) whereas three other mutants significantly decreased the survival rate (red lines) compared to that of wild-type PAO1. The degree of difference was statistically significant in both cases as determined by a log-rank analysis (*P* < 0.05). (**B**) Disc diffusion antibiograms of wild-type PAO1 and four mutants with ceftazidime are shown. Filter discs with increasing concentrations of ceftazidime (as indicated at the top) were placed on LB agar plates and inoculated with the indicated bacterial strains. After overnight growth, the cleared zones of inhibition were visualised. The numbers below disc plates indicate diameters of inhibition zone (mm).

We prioritised PAO1 genes using the pathway-centric search algorithm (see Fig. 2A) on the PseudomonasNet web server using the 38 virulence genes as input data. We selected 27 genes from the top-ranked candidates based on the availability of transposon-insertion mutants for follow-up experimental analysis. To validate whether the selected genes are involved in *P. aeruginosa* virulence, we examined the effect of each gene disruption on bacterial virulence towards *C. elegans*. We monitored the survival rate of worms (n = 90) fed with each mutant. The average lifespan of the *C. elegans* N2 worms fed PAO1 was 8.38 ± 0.40 days (Fig. 3A, black line), whereas N2 worms fed the standard *E. coli* OP50 strain lived for 11.08 ± 0.47 days (Fig. 3A, green line). Among 27 tested genes, the disruption of six genes significantly altered the survival rate of *C. elegans* N2 compared with worms fed wild-type PAO1 (*p-value* < 0.05 by log-rank test, Supplementary Table S2). Three of these genes, *PA0996* (*pqsA*), *PA0999* (*pqsD*), and *PA3478* (*rhlB*), were determined to positively regulate PAO1 virulence. The survival rate of *C. elegans* increased substantially when fed with each of these mutants (Fig. 3A, blue lines). The *pqsAD* genes are components of a five-gene operon involved in the production of Pseudomonas Quinolone Signal (PQS), a molecule that mediates *P. aeruginosa* quorum sensing (QS)^34^,^35^,^36^. The *rhlB* gene encodes a subunit of rhamnosyltransferase. Notably, the *rhlB* gene is located adjacent to *rhlR*, a gene that encodes a major QS regulator^37^. Although these genes were previously characterised to be associated with virulence, their apparent roles in the *C. elegans* infection model were not recognised in a previous genome-wide screening study^33^. It is therefore reasonable to claim that the network-assisted approach can complement genetic screening, which sometimes suffer from false negative identifications.

Disruptions of three other genes (*PA2553*, *PA3329*, and *PA3972*) resulted in elevated *P. aeruginosa* PAO1 virulence in *C. elegans* (Fig. 3A, red lines). The survival rate of *C. elegans* was significantly decreased when the worms were fed with each of these three mutants. This effect was the most significant for the *PA2553* gene mutation; worms fed this mutant had an average lifespan of less than seven days. The functional roles of these genes are not clearly defined. To search for functional clues about these new negative regulators of virulence, we employed the gene-centric search algorithm (see Fig. 2B) on the PseudomonasNet web server, in which candidate GO biological process terms are prioritised for a query gene by their enrichment among network neighbours of the query gene. Gene-centric searches for *PA2553* and *PA3972* predicted ‘phenylacetate catabolic processes’ within the top three candidate-associated pathways (Supplementary Table S3). The phenylacetate catabolic pathway has previously been reported to be required for virulence of *Burkholderia cenocepacia*, which is another opportunistic pathogen in cystic fibrosis, in the *C. elegans* host model^38^. Together, these findings suggest that *PA2553* and *PA3972* are also associated with virulence in *C. elegans* via this metabolic pathway. A gene-centric search for *PA3329* predicted ‘quorum sensing’ as the third-ranked candidate pathway and ‘phenazine biosynthetic processes’ as the eighth-ranked GO biological process term. Phenazine was previously reported as a signalling factor in the quorum sensing network of *P. aeruginosa*^39^. Thus, the gene-centric search report suggests that a mutation in *PA3329* increases virulence in the *C. elegans* host via the modulation of phenazine biosynthesis, which mediates quorum sensing. These results together demonstrate the usefulness of the gene-centric search method for the study of molecular mechanisms of the identified genes involved in clinical traits.

### PseudomonasNet predicts novel genes for antibiotic resistance

We then examined whether network-assisted interrogation can identify genes involved in antibiotic resistance, which is an important trait of *P. aeruginosa* as a major nosocomial pathogen. We employed the context-centric search algorithm (see Fig. 2C), which uses DEGs as input data, to predict genes related to antibiotic resistance. We predicted genes related to ceftazidime resistance using 325 PAO1 genes that were determined to be differentially expressed in response to treatment with ceftazidime (250 ng/mL) (*P*-value < 1.0e-5)^40^. We selected 30 genes from the top candidates by context-centric search in PseudomonasNet based on the availability of transposon-insertion mutants, and examined the effect of each gene deletion on the sensitivity to ceftazidime. Notably, four different mutants, in which *PA1556*, *PA4067*, *PA0511*, or *PA0510* gene was inactivated, exhibited elevated ceftazidime resistance. Minimal inhibitory concentration (MIC) values were increased more than 3-fold in each of these mutants when compared with the MIC of ceftazidime in the wide-type PAO1 strain (Supplementary Table S4). Consistent with this result, enhanced resistance against ceftazidime was visible on a disc diffusion assay when each of these four genes was disrupted (Fig. 3B).

We employed the gene-centric search algorithm on the PseudomonasNet web server to search for functional clues in the four novel genes involved in ceftazidime resistance (see Fig. 2B). Interestingly, all four genes were predicted to fall into the category of ‘generation of precursor metabolites and energy’ as the top candidate-associated pathways (Supplementary Table S5). *PA0510* and *PA0511* are likely involved in the biosynthesis of heme, whereas *PA1556* encodes a subunit of cytochrome oxidase. As a major outer membrane protein, OprG, which is encoded by *PA4067*, has been reported to be involved in iron uptake^41^. During the MIC test, it was observed that mutations in *PA4067*, *PA0510*, and *PA0511* genes resulted in slow bacterial growth compared with the wild-type PAO1 strain. After static overnight culture in LB, OD_600_ values of these three mutants were approximately 87% of that of the wild-type PAO1 strain (data not shown). This result further suggests that antibiotic susceptibility is closely related to bacterial growth rate^42^,^43^. More importantly, our network-assisted functional predictions yielded a set of genes that show consistent phenotypes in a given context.

### PseudomonasNet accounts for the pervasiveness of cross-resistance and provides mechanistic insights into the trade-off in resistance to different drugs

**Figure 4:**
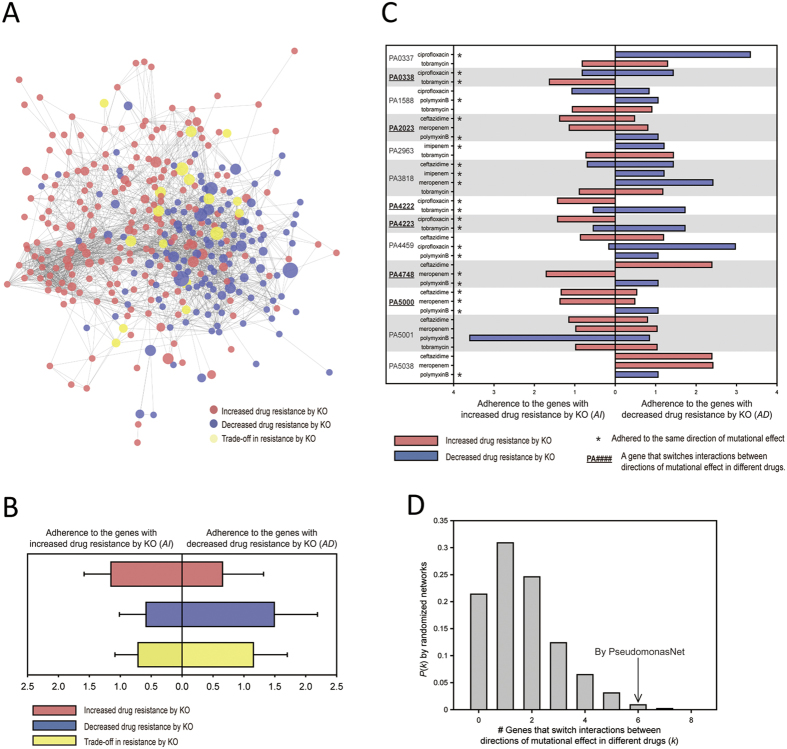
Analysis of cross-resistance and trade-offs in resistance to different antibiotics using an antibiotic resistance network. (**A**) A network of 339 antibiotic-resistant genes based on PseudomonasNet is shown. Blue nodes represent genes in which antibiotic resistance is decreased by knockout (i.e., positive regulation of antibiotic resistance) and red nodes represent genes in which antibiotic resistance in increased by knockout (i.e., negative regulation of antibiotic resistance). Yellow nodes represent genes that show a trade-off in resistance to different antibiotics. The node size is proportional to the number of antibiotics whose resistance are changed by perturbation of the gene. (**B**) Adherence of the genes with each direction of mutational effect to the same directional group of genes in PseudomonasNet. Box and error bars represent distribution of mean adherence score and standard deviation, respectively, for each group of genes. (**C**) The normalised connection scores to two groups of genes for different directions of mutational effect are represented as bars projecting in opposite directions: right for those that decrease antibiotic resistance by knockout and left for those that increase antibiotic resistance by knockout. If the given gene interacts with other genes with the same direction of mutational effect for each drug, then the blue bars are expected to project to the right and the red bars are expected to project to the left. The asterisk symbol (*) represents genes that exhibit adherence to the same direction of mutational effect. Genes that are underlined were shown to switch their interactions between directions of mutational effect in different antibiotic treatments. (**D**) The distribution of the number of genes that switch their interactions between directions of mutational effect under different antibiotic conditions for 1,000 randomised networks is shown.

In order to extend our network-assisted investigation to the antibiotic-resistance system, we constructed a network of antibiotic-resistant genes against multiple drugs based on PseudomonasNet. A total of 372 PAO1 genes involved in the regulation of resistance against six different antibiotics were collected from previous studies: ceftazidime^44^, ciprofloxacin^45^, imipenem^44^, meropenem^44^, polymyxin B^46^, and tobramycin^47^,^48^ (Supplementary Table S6). The direction of mutational effect in antibiotic resistance exerted by each gene was determined based on the change in drug resistance following disruption of the gene. The inactivation of each gene resulted in either increased or decreased drug resistance, which suggests that each gene regulates drug resistance in either direction. The genes that are determined to affect each drug resistance in each direction are modular and highly predictive in PseudomonasNet, as indicated by the high AUC scores (Supplementary Table S6). We also included four newly identified genes whose mutation increase ceftazidime resistance, *PA1556*, *PA4067*, *PA0511*, or *PA0510*, into our investigation of antibiotic-resistance system. PseudomonasNet connects 339 unique antibiotic-resistant genes into the largest component network, which will be referred to as the ‘antibiotic-resistance network’ below. We observed that the antibiotic-resistance network is partitioned into two network communities, each corresponding to a direction of mutational effect to drug resistance: genes whose inactivation results in increased resistance (red) and genes whose inactivation results in decreased resistance (blue) (Fig. 4A). The node size is proportional to the number of antibiotics whose resistance is affected by perturbation of the gene. To conduct a more quantitative analysis of the modularity of genes for each direction of mutational effect on drug resistance, we devised a score to measure the adherence to either group of genes (see Methods for details). We confirmed that antibiotic-resistant genes are significantly more adherent to other genes with the same direction of mutational effect (*P* = 6.49e-11 and *P* = 4.29e-13 for genes with increased and decreased drug resistance by knockout, respectively; Wilcoxon signed rank test, Fig. 4B). These results suggest that the antibiotic resistance systems of *P. aeruginosa* have modular genetic organisations for individual drugs as well as for each direction of mutational effect on resistance, which accounts for the prevalence of cross-resistance, in which the knockout of a gene affects the resistance to multiple drugs with the same direction of effect.

The antibiotic resistance network also includes 17 genes that participate in both directions of mutational effect (yellow nodes of the network in Fig. 4A), showing insignificant adherence to both directions of mutational effect (*P* = 0.1075 by Wilcoxon signed rank test, Fig. 4B). The direction of mutational effect of these genes depends on the antibiotic that is used for treatment. We categorised these genes as those involved in the trade-off in resistance. Interestingly, the genes that show this trade-off in resistance are located between the two network communities for the two directions of mutational effect. Based on this network topology, we hypothesised that these genes change their directions of mutational effect for different drugs by switching interaction partners between the two groups of genes, whose mutations decrease resistance and those increase resistance. To test this hypothesis, we analysed the interaction-bias of these 17 genes towards genes for the same direction of mutational effect under different drug conditions (see Methods for details). We found that 13 of these 17 genes have connections to both groups of genes in the network of 339 antibiotic-resistant genes. From the interaction-bias analysis, we found that six (*PA0338, PA2023, PA4222, PA4223, PA4748*, and *PA5000*) of these 13 genes (46%) switch their interaction-bias towards the same direction of mutational effect between different drug conditions (Fig. 4B), which is a significant observation compared with those by randomised networks (*P-value* = 0.01 by permutation test using 1,000 randomised networks) (Fig. 4C). These results demonstrate that PseudomonasNet can facilitate the study of the underlying biology for the resistance of *P. aeruginosa* to different antibiotics.

## Discussion

The genetic system of the human opportunistic bacterial pathogen *P. aeruginosa* is highly complex, which enables *P. aeruginosa* to be robust and adaptable under many host and drug conditions. Functional gene network models have been utilised to facilitate the genetic dissection of complex traits such as human diseases^49^. Although bacteria are single-celled organisms, screening for their virulence and antibiotic resistance generally uncovers many associated genes. Moreover, the subset of these genes that form the network for clinically important traits varies across different infection conditions. To explore the large genetic search space for pathogenicity and antibiotic resistance in *P. aeruginosa*, a research platform for the systematic dissection of the genetic components of complex traits is needed. In this study, we presented a genome-scale functional network of *P. aeruginosa* genes, and demonstrated the feasibility of network-assisted gene identification for virulence and antibiotic resistance with experimental validation. We used two different network-assisted search algorithms to predict candidate genes for clinically important traits: the pathway-centric search, which starts with known genes for a pathway or trait, and the context-centric search, which starts with DEGs for a clinical condition. We achieved ~22% (6/27) and ~13% (4/30) discovery rates for genes involved in virulence within the *C. elegans* host and ceftazidime resistance, respectively. These discovery rates are ~32-fold and ~13-fold more effective than unbiased genome-wide screens for the genes involved in virulence within *C. elegans* (38/5572 = 0.68%) and ceftazidime resistance (55/5572 = 0.99%), respectively. Therefore, if some genes for virulence have already been identified from initial unbiased genome-wide screens, then it would be more effective to experimentally test only the candidates predicted from the pathway-centric search option of the web server using the seed genes identified from the screen than to repeat the same genome-wide screen. Similarly, if we have gene expression data for a condition related to a clinical trait, then the context-centric search would be a cost-effective approach for the next round of screen.

PseudomonasNet revealed the functional communities of genes for each direction of mutational effect on antibiotic resistance across multiple drugs. Thus, the antibiotic resistance network suggest that direction of mutational effect on drug resistance is regulated by pathways rather than individual genes, and explains the frequently observed cross-resistance of *P. aeruginosa* genes to multiple drugs via the high functional coherence for each direction of mutational effect. In addition, PseudomonasNet provides mechanistic insights into the trade-off in resistance to different drugs by showing a switch in the interaction-bias towards genes with the same direction of mutational effect on different drugs. These results demonstrate the value of genome-scale functional networks to study the underlying mechanisms of multi-drug resistance in pathogenic microbes.

Functional networks map co-functional relationships between genes, which do not necessarily indicate specific underlying mechanisms for the functional associations. For example, co-cited genes are likely to be functionally coupled, albeit no clue whether they interact directly or indirectly. Functional networks are therefore inherently limited for the study of underlying molecular interactions for the phenotypes such as bacterial pathogenicity. However, as demonstrated in this study, combined use of the functional network in prioritizing candidate genes and loss-of-function analysis will accelerate discovery of new genetic components of bacterial pathogenicity. Investigation of specific molecular mechanisms of their involvement in the pathogenicity may need additional computational and experimental tools.

Recently, studies of the molecular evolution of *P. aeruginosa* that have sequenced bacterial clones isolated from patients have identified genes that show parallel evolution; these genes are suggested to be critical to host adaptation^50^. This sequencing-based approach has been applied to cancer genomes, which has uncovered several cancer gene candidates based on the mutation frequency among patients. However, these studies have revealed that somatic mutations in cancer genes occur in only a minority of patients^51^, which suggests that the ability to identify cancer genes from mutational information is limited. A sequencing-based approach to study clinically important traits such as the virulence and drug resistance of bacterial pathogens in patients may suffer from similar limitations in the future. Cancer genomics now employ pathway and network approaches to analyse somatic mutation data derived from patients^52^. Similarly, the analysis of mutation data from pathogenic bacterial strains isolated from patients could benefit from these pathway and network approaches. For example, identification of subnetworks enriched for mutations among drug resistance strains may reveal genes or pathways that drives antibiotic resistance. Thus, genome-scale functional networks for these pathogenic microbes, such as PseudomonasNet, may be a useful resource for pathway and network approaches in the future analysis of clinical microbial genomics data.

*P. aeruginosa* is a versatile organism with a robust capability to adapt to diverse growth conditions. Microarray-based whole-genome typing indicates that *P. aeruginosa* strains, regardless of whether they are recovered from the environment or a patient, possess a highly conserved genome^53^. Inside the airway mucus layer of patients with cystic fibrosis, *P. aeruginosa* strains with mutations in the *mucA* gene, which encodes an anti-sigma factor^1^, and *lasR*, a gene involved in QS^54^, have been isolated. Together, these results suggest that *P. aeruginosa* can increase its survival fitness by selectively acquiring or losing only a small number of regulatory genes rather than by a larger degree of genome rearrangement. PseudomonasNet will be useful to explore the physiological consequences of a defined gene mutation, which may be detected in certain clinical isolates from patients with significant disease symptoms.

PseudomonasNet has proved its prediction power as described in the present study. Massive amount of genomics data generated by next generation sequencing in coming years can be incorporated into the current network by retraining and will potentially improve predictions. We anticipate that PseudomonasNet will accelerate the functional annotation of unknown genes as well as expand our understanding of clinically important traits of *P. aeruginosa*, which may lead to the development of novel antibiotics or anti-virulence therapies in the future.

## Methods

### Sequences and functional annotation data for *Pseudomonas aeruginosa*

The genome sequence and 5,572 protein-coding genes for *P. aeruginosa* PAO1 were downloaded from the Pseudomonas Genome Database^55^, and the reference functional annotation data for *P. aeruginosa* were downloaded from Gene Ontology^23^.

### Construction of PseudomonasNet

Co-functional links were inferred from nine distinct data types (see Table 1) using machine learning methods, and then integrated into PseudomonasNet using a Bayesian statistical framework. A detailed description of the network construction is provided in the Supplementary Online Methods.

### Network visualisation and centrality analysis

All network visualisations are performed on Cytoscape software^56^ with the organic layout option. The degree centrality for gene *t* represents the number of genes that have a direct link with gene *t.* The betweenness centrality for gene *t* (*B*_*t*_) is calculated as:


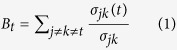


In eq. (1), *σ*_*jk*_ represents the number of shortest paths between node *j* and node *k*, and *σ*_*jk*_(*t*) represents the number of shortest paths between node *j* and node *k* that pass through node *t*. The edge weight score is ignored when calculating the betweenness centrality.

### Adherence score and interaction-bias analysis

For the given network of genes involved in antibiotic resistance, we calculated the adherence of gene *t* to genes with increased or decreased resistance to drug *d* by knockout (*AI*_*d*_(*t*) *or AD*_*d*_(*t*) respectively) by the following equations:


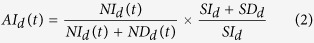



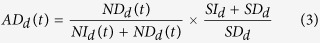


In eqs (2) and (3), *NI* and *ND* represent the number of neighbours with increased and decreased resistance by knockout, respectively, and *SI* and *SD* represent the number of genes with increased and decreased resistance by knockout, respectively. For a given drug *d*, naïve adherence scores 
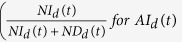
 and 
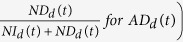
 need to be normalized by the ratio of *SI* and *SD* 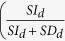 and 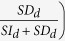, respectively, to account for the difference in the total number of antibiotic resistance genes among drugs.

We determined whether gene *t* switches interactions between directions of mutational effect in different drugs using the following criteria: i) if *t* increases the resistance to *d* by knockout and 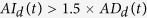 or ii) if *t* decreases the resistance to *d* by knockout and 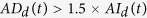.

### Virulence test in *C. elegans*, MIC determination, and disc diffusion assay

Virulence tests were performed following procedures that have been described previously^57^. In brief, 10 μl each of overnight-grown bacterial culture was spotted on Nematode Growth Medium (NGM) agar plates. After incubation for 2 h at room temperature, each plate was seeded with 10 adult hermaphrodite worms (nine replicates per trial) and incubated at 20 °C. Viability of worms was monitored every 24 hr and live worms were transferred to fresh NGM plates every 48 hr to exclude the newborn larvae. PAO1 was used as a control. The MIC test and disc diffusion assay were performed as described previously^58^.

## Additional Information

**How to cite this article**: Hwang, S. *et al.* Network-assisted investigation of virulence and antibiotic-resistance systems in *Pseudomonas aeruginosa*. *Sci. Rep.* **6**, 26223; doi: 10.1038/srep26223 (2016).

## Supplementary Material

Supplementary Information

## Figures and Tables

**Table 1:** Nine component networks incorporated into PseudomonasNet.

Code	Description	# of genes	# of links
PA-CC	Links inferred by co-citation across PubMed central articles for *P. aeruginosa*	2,673	72,947
PA-CX	Links inferred by co-expression across gene expression profiles of the GEO database	4,271	49,483
PA-DP	Links inferred by the correlation of protein domain profiles of *P. aeruginosa* coding genes	2,589	5,000
PA-GN	Links inferred by the neighbourhood of *P. aeruginosa* orthologues in bacterial genomes	4,022	32,000
PA-PG	Links inferred by the correlation of phylogenetic profiles of *P. aeruginosa* genes	2,031	31,496
EC-CC	Associalogs of links inferred by co-citation across PubMed central articles for *E. coli* biology	1,403	26,484
EC-CX	Associalogs of links inferred by co-expression across *E. coli* gene expression profiles of GEO database	1,999	37,483
BA-HT	Associalogs of bacterial protein-protein interactions derived from high-throughput analysis	1,768	12,361
BA-LC	Associalogs of bacterial protein-protein interactions derived from the literature curation of small-scale analyses	626	893
PseudomonasNet	Integrated network of the nine component networks	5,456	203,118

PA, *P. aeruginosa*; EC, *E. coli*; BA, other bacteria; CC, co-citation; CX, co-expression; DP, domain profiling method; GN, gene neighbourhood method; PG, phylogenetic profiling method; HT, protein-protein interactions by high-throughput assay; LC, literature-curated protein-protein interactions.
