# Supplementary material for: Network-assisted investigation of virulence and antibiotic-resistance systems in Pseudomonas aeruginosa
Source: Sci Rep. 2016 May 19;6:26223. doi: 10.1038/srep26223 (PMC4872156; doi:10.1038/srep26223)
Supplement: Supplementary Information [file srep26223-s1.pdf]

Supplementary Online Information

Network-assisted investigation of virulence and antibiotic-resistance systems in *Pseudomonas aeruginosa*

Sohyun Hwang, Chan Yeong Kim, Sungou Ji, Junhyeok Go, Hanhae Kim, Sunmo Yang, Hye Jin Kim, Ara Cho, Sang Sun Yoon, and Insuk Lee

☐ **Contents**

- **Supplementary Online Methods**
- **Supplementary Figure S1**
- **Supplementary Figure S2**
- **Supplementary Figure S3**
- **Supplementary Table S1**
- **Supplementary Table S2**
- **Supplementary Table S3**
- **Supplementary Table S4**
- **Supplementary Table S5**
- **Supplementary Table S6**
- **Supplementary Table S7**
- **Supplementary References**

Supplementary Online Methods

Gold standard co-functional gene pairs

Network links were inferred from various data using supervised learning approach, which requires gold standard data to train input data. To generate positive gold standard of co-functional gene pairs, we paired two genes annotated for same pathways, assuming they are functionally coupled. For the training *P. aeruginosa* gene functional links, we used biological process annotations by Gene Ontology (as of April 16th, 2012). Both accuracy and size of gold standard data are important for training. Therefore, we empirically selected three GO evidences, IDA (inferred from direct assay), ISS (inferred from sequence and structural similarity), and RCA (inferred from reviewed computational analysis). We also generated negative gold standard gene pairs by pairing genes annotated for different GO terms supported by the same three evidences. The resultant gold standard set has 16,062 positive and 393,903 negative gene pairs among 906 *P. aeruginosa* genes.

Benchmarking and Bayesian data integration

The likelihood of co-functional link supported by given experimental or computational data was measured by Bayesian statistic approach.[1] Log likelihood score (LLS) was calculated by following equation:

$$LLS = \ln(\frac{P(L|E)/P(\neg L|E)}{P(L)/P(\neg L)})$$

, where $P(L|E)$ and $P(\neg L|E)$ are probability of gold standard positive and negative link for given experimental data, respectively. $P(L)$ and $P(\neg L)$ are probability of gold standard positive and negative link before the experimental data provided, respectively. For data sets in which each gene pair is associated with a continuous score (e.g., correlation coefficient), we calculated LLS scores for bins containing equal numbers of gene pairs using gold standard gene pairs. Those LLS scores and their corresponding data scores (the mean data scores for a bin) were used to calculate regression models, which were then used to map individual data intrinsic scores to LLS scores for both gold standard gene pairs and unlabeled gene pairs in a continuous manner. Finally, we take only gene pairs that are significantly higher likelihood than those by random chance (e.g., LLS > 0.7 or 2 fold more likely than random chance).

Since we inferred functional associations from nine distinct data sets (**Table 1**), a functional association between genes could be supported by multiple LLSs that pass the cutoff. For the links with multiple LLSs, we integrated the scores using weighted sum (WS) method as described in [2]:

$$WS = L_0 + \sum_{i=1}^{n} \frac{L_i}{D \cdot i}, \; for\ all\ L \geq T$$

, where L represents LLS (L_0 is the maximum LLS of a given functional link), and i is the index number for all other LLS by ranked order. D is a free parameter used as a weight factor, and T is a minimum threshold of LLS. If data types to be integrated are not correlated at all, summation of all scores (e.g., naïve Bayes) would be the optimal method for integration. In contrast, if the data types are completely correlated, taking the maximum of the scores would result in the best integration. However, data types in

general have partial correlation, in which taking partial credit of additional score with appropriate weight improves the integrated network. We choose the free parameters for the weighted sum where we achieve the best precision-recall performance of the integrated network.

Co-functional links inferred from co-citation of *P. aeruginosa* genes (CC)

The original co-citation algorithm was based on an idea that functionally related two *P. aeruginosa* genes tend to be cited at the same research article abstract.[3] However, some articles have names of genes in the main text. To improve sensitivity of search, we search PubMed Central (PMC, http://www.ncbi.nlm.nih.gov/pmc/) for articles containing "Pseudomonas aeruginosa" in abstract and any *P. aeruginosa* gene name in full text. As a result, we found a total of 8,029 articles containing *P. aeruginosa* gene names, and then assign probability of association between genes by one-tail Fisher's exact test.

Co-functional links inferred from co-expression of *P. aeruginosa* genes (CX)

We downloaded 34 microarray data sets containing no less than 8 samples of gene expression from Gene Express Omnibus (GEO)[4] on October 30th, 2012. Pearson correlation coefficient was measured between all pairs of gene vectors of expression values to infer functional association between two genes. We tested a total of 34 microarray data sets and were able to infer functional links from 12 of them (**Supplementary Table S7**).

Co-functional links inferred from correlation of protein domain profiles (DP)

Domains are recurring functional motifs of proteins. Because domains are structural, functional and evolutionary units of protein, proteins that share a similar set of domains are likely to be functionally associated. Using profiles of domain occurrence in proteins by InterPro database,[5] we measure likelihood of functional association for given tendency of domain co-occurrence between two proteins. To learn a more informative co-occurrence pattern, we used a weighted version of mutual information score, in which higher weights were given to more infrequent domains under the assumption that infrequent domains harbor specific pathway information.

Co-functional links inferred from genomic contexts (PG and GN)

We used genomic context information of *P. aeruginosa* genes to discover functional associations between genes with two different methods, phylogenetic profiling[6] and gene neighborhood.[7] The similarity of phylogenetic profiles between two *P. aeruginosa* genes reflects the degree of co-inheritance of two genes during speciation, because functional constraints between functionally coupled genes mainly determine co-inheritance pattern. We first ran BLASTP to compare all *P. aeruginosa* protein sequences against all protein sequences from 1,626 bacteria genomes, 122 archaea genomes and 396 eukaryotic genomes. Phylogenetic profile matrices of the blast-hit scores were constructed and the association between profiles was measured by mutual information scores. For *P. aeruginosa* genes, we found that the similarity of phylogenetic profiles for each of the two domains of life (archaea and bacteria)

performed better than that for all 2,144 genomes in retrieving gold-standard functional links. We integrated the two domain-specific networks into a single network by phylogenetic profiles.

For network inference by genomic neighborhood across 1,746 prokaryote genomes, we used two approaches to measure the genomic neighborhood, distance-based gene neighbourhood (DGN) and probability-based gene neighbourhood (PGN), as described in our previous work[7]. For the DGN measure, we took the median value of chromosomal distance between orthologs of the two query genes with PBLAST E-values < 1 across the 1,746 reference prokaryote genomes. Each median distance value was normalized using the number of genomes in which orthologs of the two genes co-occurred, giving greater weight to gene pairs conserved in a larger number of prokaryote genomes. For the PGN measure, we calculated the probability of two genes being separated by fewer than *d* genes in a genome containing *N* genes as follows:

$$P(\leq d) = \frac{2d}{N-1}$$

We then calculate the product of the above probability across the *m* reference genomes containing orthologs of the two query genes:

$$X = \prod_{i=1}^{m} P_i(\leq d_i) = \prod_{i=1}^{m} \frac{2d_i}{N_i - 1}$$

To calculate the likelihood that two genes belong to the same conserved neighbourhood, we determine the probability of obtaining a value of *X* that is smaller than the observed value:

$$P_m(\leq X) = 1 - P_m(> X) \approx X \sum_{k=0}^{m-1} \frac{(-lnX)^k}{k!}$$

Because we previously found complementarity between these two methods, we integrated them into a single network by genomic neighborhood using weighted sum method as described above.

Co-functional links by orthology-based transfer from *Escherichia coli* and bacterial protein-protein interactions

Associalogs are conserved functional associations transferred from different species by orthology.[8] We transferred conserved functional links between *P. aeruginosa* genes from *E. coli* and bacterial protein-protein interactions. All transferred co-functional associations are re-scored by Inparanoid weighted *LLS* (*IWLLS*)[8] as following:

IWLLS (A'-B') = *LLS* (A-B) + ln(inparalog score of A-A') + ln(inparalog score of B-B'),

where A' and B' are *P. aeruginosa* genes and A and B are orthologous genes from *E. coli* or other bacteria, and the transferred functional association of A'-B' from that of A-B obtains weighted values as how likely A-A' and B-B' are orthologous by Inparanoid.[9] We transferred our previously published *E. coli* co-citation (EC-CC) and co-expression (EC-CX) networks[10] and bacterial protein-protein interactions (BA-HT and BA-LC). BA-LC network is based on PPIs derived from literature curated small-scale experiments. These interactions were collected from four databases of BIND,[11] DIP,[12] IntAct,[13] and MPIDB.[14] We collected PPIs of *E. coli* and *H. pylori* as well as

those of *P. aeruginosa*, because *P. aeruginosa* PPIs are very limited. BA-HT network is based on high-throughput PPI assays, collected from six high-throughput yeast two-hybrid experiments for *H. pylori,*[15] *Treponema pallidum,*[16] *Synechocystis sp. PCC6803,*[17] *Mesorhizobium loti,*[18] *Campylobacter jejuni,*[19] and *E. coli*[20] and from four high-throughput tandem affinity purification followed by mass spectrometry analysis for *E. coli*[21-23] and *M. pneumonia.*[24] Then, these bacterial PPI networks were converted into *P. aeruginosa* protein networks by the associalog method as described above.

Evidence	PA-PG	PA-GN	PA-DC	PA-CX	PA-CC	EC-CX	EC-CC	BA-LC
BA-HT	0.11	0.33	0.01	0.19	0.19	0.93	1.12	0.10
BA-LC	0.06	0.08	0.00	0.03	0.10	0.12	0.21	
EC-CC	0.61	1.06	0.05	0.44	1.10	3.04		
EC-CX	0.53	0.94	0.02	0.63	0.76			
PA-CC	0.43	0.95	0.21	3.18				
PA-CX	0.18	0.57	0.06					
PA-DC	0.05	0.01						
PA-GN	1.04							

Supplementary Figure S1. Pairwise overlap analysis between nine component networks of PseudomonasNet. The number of each cell indicates the percentage of the number of PseudomonasNet links. The percentage of overlaps are also indicated by color code, red for higher overlap and green for lower one. The largest number of overlap links is observed between PA-CC (Pseudomonas gene co-citation links) and PA-CX (Pseudomonas gene co-expression links), which still account for only 3.18% of all links of PseudomonasNet.

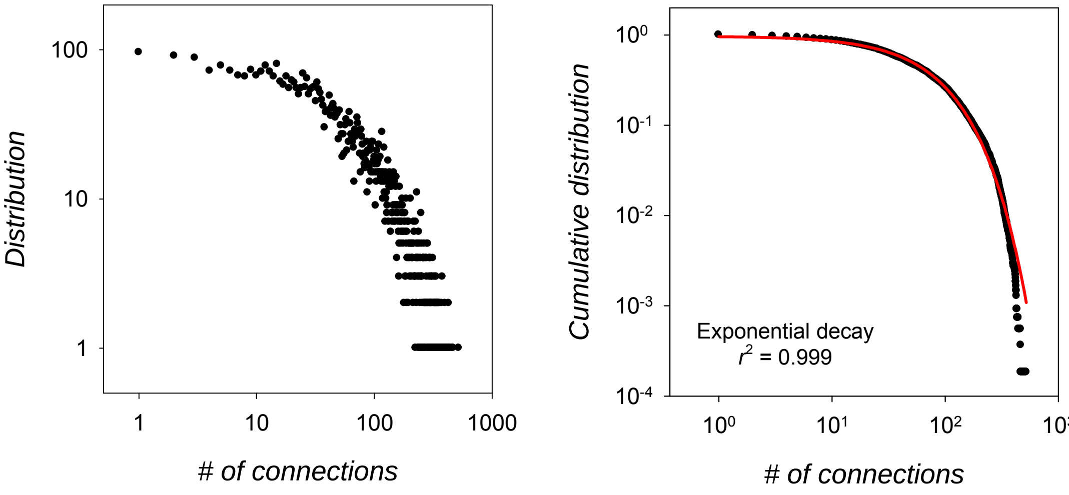

Supplementary Figure S2. Degree distribution of PseudomonasNet. Distribution of the number of connections indicates that PseudomonasNet is a small-world network with broad-scale, which is characterized by a connectivity distribution that has a power law regime followed by a sharp cutoff such as exponential decay of the tail (r^2 = 0.999). The similar degree distribution were observed from task-driven social networks (e.g., Board of directors) and other functional gene networks.

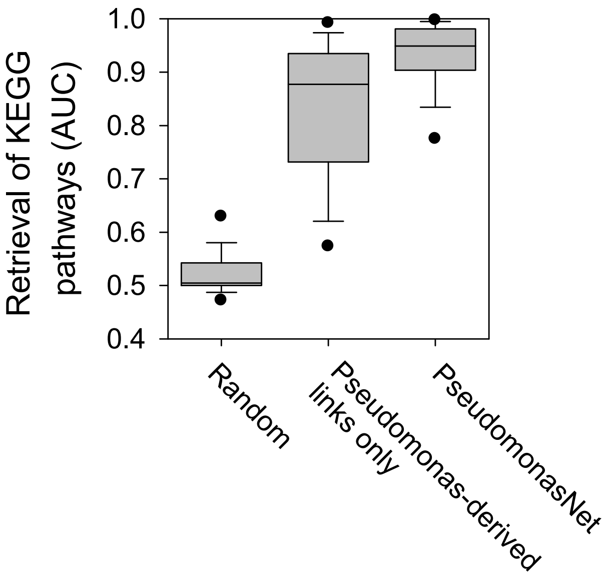

Supplementary Figure S3. Assessment of PseudomonasNet prediction for KEGG pathways. Area under ROC curve (AUC) for retrieval of member genes for each of 100 KEGG pathways with no less than four members were measured. We also excluded nine KEGG terms for global and overview maps, which is for relatively broad pathway concepts such as ‘metabolic pathways’. To test contribution of the functional links derived from Pseudomonas data (PA-CC, PA-CX, PA-DP, PA-GN, PA-PG) in KEGG pathway prediction, we constructed a network with no links supported exclusively by other bacterial data, resulted in a network of 157,395 links (~77.5% of all links of PseudomonasNet). The distribution of AUC for 100 KEGG pathways indicates that PseudomonasNet is highly predictive for KEGG pathways and Pseudomonas data made significant contribution to the pathway prediction.

Supplementary Table S1. 38 *P. aeruginosa* virulence genes in *C. elegans*

ORF_ID	Symbol	PA14 ortholog	*P. aeruginoa* GO biological process
PA0337	ptsP	PA14_04410	transport; phosphoenolpyruvate-dependent sugar phosphotransferase system
PA0407	gshB	PA14_05310	cellular amino acid metabolic process;glutathione biosynthetic process; cofactor biosynthetic process
PA0456	na	PA14_05960	na
PA0652	vfr	PA14_08370	regulation of transcription, DNA-dependent
PA0745	na	PA14_54640	na
PA0795	prpC	PA14_53950	fermentation;metabolic process; cellular catabolic process
PA0796	prpB	PA14_53940	fermentation;metabolic process; cellular catabolic process; cellular lipid metabolic process
PA0895	aruC	PA14_52720	cellular amino acid metabolic process; arginine biosynthetic process
PA0897	aruG	PA14_52690	cellular amino acid metabolic process; arginine catabolic process
PA0898	aruD	PA14_52670	cellular amino acid metabolic process
PA0899	aruB	PA14_52660	cellular amino acid metabolic process
PA0904	lysC	PA14_52580	cellular amino acid metabolic process; lysine biosynthetic process via diaminopimelate
PA0928	gacS	PA14_52260	two-component signal transduction system (phosphorelay)
PA1000	pqsE	PA14_51380	cofactor biosynthetic process
PA1216	na	PA14_48590	na
PA1432	lasI	PA14_45940	response to stimulus
PA1592	na	PA14_43900	na
PA1636	kdpD	PA14_43350	two-component signal transduction system (phosphorelay)
PA1665	fha2	PA14_42950	na
PA1766	na	PA14_41730	na
PA1767	na	PA14_41710	na
PA2015	liuA	PA14_38440	cellular catabolic process
PA2550	na	PA14_31580	na
PA2586	gacA	PA14_30650	regulation of transcription, DNA-dependent
PA2591	vqsR	PA14_30580	na
PA2620	clpA	PA14_30230	cellular protein metabolic process
PA2965	fabF1	PA14_25690	cellular lipid metabolic process
PA3244	minD	PA14_22020	cytokinesis by binary fission
PA3477	rhlR	PA14_19120	regulation of transcription, DNA-dependent; response to stimulus
PA3805	pilF	PA14_14850	cellular component movement; pilus assembly; secretion

PA4005	na	PA14_12030	na
PA4222	na	PA14_09320	na
PA4223	na	PA14_09300	na
PA4664	hemK	PA14_61680	na
PA5203	gshA	PA14_68730	cellular amino acid metabolic process; glutathione biosynthetic process; cofactor biosynthetic proces
PA5224	pepP	PA14_69000	proteolysis; cellular protein metabolic process
PA5288	glnK	PA14_69810	regulation of nitrogen utilization; metabolic process
PA5484	kinB	PA14_72390	na

Supplementary Table S2. Six novel *P. aeruginosa* virulence genes in *C. elegans* survival analysis.

Strains	Average survival time in day (mean ± SEM)	*p-value* of difference from PAO1 in survival time
PAO1	8.38 ± 0.40	-
Δ*PA0999*	9.76 ± 0.43	0.021
Δ*PA0996*	9.64 ± 0.43	0.036
Δ*PA3478*	9.60 ± 0.50	0.025
Δ*PA3329*	7.24 ± 0.38	0.048
Δ*PA3972*	7.13 ± 0.33	0.011
Δ*PA2553*	6.96 ± 0.32	0.004

*A total of 90 worms were used in each experiment. Average survival time was calculated by the Kaplan Meier algorithm and presented with standard error of the mean (SEM). P-values were calculated by the log-rank test. We selected six mutants that show significant difference from PAO1 in survival time (*p-value* < 0.05)

Supplementary Table S3. Predicted GO biological process terms by gene-centric search of PseudomonasNet for three novel *P. aeruginosa* genes that increase virulence in *C. elegans* by knockout mutant

A. PA2553

Rank	Score	GO biological process term
1	23.92	cellular catabolic process(PA)
2	13.30	response to DNA damage stimulus(EC)
3	12.33	phenylacetate catabolic process(EC)
4	9.12	cofactor biosynthetic process(PA)
5	6.93	generation of precursor metabolites and energy(PA)
6	6.82	anaerobic respiration(EC)
7	6.63	leucine catabolic process(PA)
8	6.63	isoleucine catabolic process(PA)
9	6.63	valine catabolic process(PA)
10	6.00	carnitine metabolic process(EC)

B. PA3972

Rank	Score	GO biological process term
1	18.67	response to DNA damage stimulus(EC)
2	11.59	phenylacetate catabolic process(EC)
3	9.53	transcription, DNA-dependent(EC)
4	8.31	response to oxidative stress(EC)
5	7.87	response to stress(EC)
6	6.92	cellular catabolic process(PA)
7	6.74	DNA dealkylation involved in DNA repair(EC)
8	6.33	carnitine metabolic process(EC)
9	4.91	response to cold(EC)
10	4.52	transcription antitermination(EC)

C. PA3329

Rank	Score	GO biological process term
1	20.77	cofactor biosynthetic process(PA)
2	13.22	response to stimulus(PA)

3	10.52	quorum sensing(PA)
4	10.21	secretion(PA)
5	9.77	generation of precursor metabolites and energy(PA)
6	9.39	regulation of transcription, DNA-dependent(PA)
7	8.29	transport(PA)
8	6.73	phenazine biosynthetic process(PA)
9	6.34	response to DNA damage stimulus(EC)
10	6.13	cellular catabolic process(PA)

Supplementary Table S4. Four novel PAO1 genes for ceftazidime resistance

Rank	Mutated gene	MIC (ug / ml)			
		Day1	Day2	Day3	Average
CTRL	Wild type	1	1	1	1
3	PA1556 (ccoO2)	4	4	2	**3.33**
6	PA4067 (oprG)	8	8	8	**8**
22	PA0511 (nirJ)	4	8	4	**5.33**
26	PA0510	8	4	8	**6.66**

Supplementary Table S5. Predicted GO biological process terms by gene-centric search of PseudomonasNet for the four novel *P. aeruginosa* genes that increase resistance against ceftazidime by knockout mutant.

A. **PA0510**

Rank	Score	GO biological process term
1	29.85	generation of precursor metabolites and energy(PA)
2	25.51	cofactor biosynthetic process(PA)
3	18.12	anaerobic respiration(EC)
4	15.27	response to DNA damage stimulus(EC)
5	13.85	cellular amino acid metabolic process(PA)
6	8.45	metabolic process(PA)
7	7.14	transport(PA)
8	7.11	heme biosynthetic process(PA)
9	6.21	heme biosynthetic process(EC)
10	5.22	regulation of transcription, DNA-dependent(PA)

B. PA0511 (nirJ)

Rank	Score	GO biological process term
1	30.91	generation of precursor metabolites and energy(PA)
2	15.91	anaerobic respiration(EC)
3	15.20	cofactor biosynthetic process(PA)
4	11.31	cellular amino acid metabolic process(PA)
5	11.30	transport(PA)
6	8.27	response to stimulus(PA)
7	7.43	metabolic process(PA)
8	6.80	response to DNA damage stimulus(EC)
9	4.59	secretion(PA)
10	4.50	regulation of transcription, DNA-dependent(PA)

C. PA1556 (ccoO2)

Rank	Score	GO biological process term
1	14.35	generation of precursor metabolites and energy(PA)
2	10.49	response to DNA damage stimulus(EC)
3	9.55	cofactor biosynthetic process(PA)

4	4.01	regulation of transcription, DNA-dependent(PA)
5	3.85	anaerobic respiration(EC)
6	3.82	response to stimulus(PA)
7	3.57	response to hydrogen peroxide(EC)
8	3.54	response to oxidative stress(EC)
9	3.39	secretion(PA)
10	3.39	protoporphyrinogen IX biosynthetic process from glutamate(EC)

D. PA4067 (oprG)

Rank	Score	GO biological process term
1	16.23	generation of precursor metabolites and energy(PA)
2	13.02	transport(PA)
3	7.98	response to stimulus(PA)
4	7.68	cofactor biosynthetic process(PA)
5	7.17	response to DNA damage stimulus(EC)
6	6.13	cellular amino acid metabolic process(PA)
7	5.24	regulation of transcription, DNA-dependent(PA)
8	4.98	transport(EC)
9	4.84	aerobic respiration(EC)
10	3.72	cellular component movement(PA)

Supplementary Table S6. PAO1 genes involved in regulation of resistance against six antibiotics

*AUC was not determined (ND) for sets with less than 10 member genes.

PMID:20679510		PMID:20679510		PMID:23070157	
Ceftazidime susceptibility increase by KO mutation (AUC=0.74)	Ceftazidime susceptibility decrease by KO mutation (AUC=0.73)	Meropenem susceptibility increase by KO mutation (AUC=0.72)	Meropenem susceptibility decrease by KO mutation (AUC=0.64)	PolymyxinB susceptibility iIncrease by KO mutation (AUC=0.88)	PolymyxinB susceptibility decrease by KO mutation (AUC: ND)
PA0011	PA0479	PA0401	PA0667	PA0401	PA1180
PA0401	PA0667	PA0420	PA0807	PA0402	
PA0402	PA0908	PA0427	PA0908	PA1375	
PA0420	PA1348	PA0770	PA0958	PA1588	
PA0427	PA2023	PA2615	PA1348	PA1799	
PA0503	PA2487	PA3050	PA2023	PA2023	
PA0766	PA2621	PA3818	PA2621	PA3050	
PA0770	PA2797	PA4005	PA3520	PA4020	
PA1011	PA3141	PA4069	PA3704	PA4069	
PA1195	PA3145	PA4269	PA3721	PA4109	
PA1483	PA3247	PA4753	PA4402	PA4459	
PA2128	PA3259	PA5288	PA4748	PA4748	
PA2615	PA3520		PA5000	PA4776	
PA2970	PA3589		PA5001	PA5000	
PA3050	PA3620		PA5003	PA5001	
PA3433	PA3667		PA5038	PA5038	
PA3649	PA3704		PA5192	PA5199	
PA3800	PA3721				
PA3818	PA4109				
PA4088	PA4402				
PA4269	PA4459				
PA4753	PA4527				
PA5288	PA4550				
	PA4748				
	PA4946				
	PA5000				
	PA5001				
	PA5002				
	PA5003				
	PA5005				
	PA5038				
	PA5443				

PMID:18824609		PMID:20679510		PMID:21253457	PMID:18824604
Ciprofloxacin susceptibility increase by KO mutation (AUC=0.82)	Ciprofloxacin susceptibility decrease by KO mutation (AUC=0.83)	Imipenem susceptibility increase by KO mutation (AUC=0.74)	Imipenem susceptibility decrease by KO mutation (AUC: ND)	Tobramycin susceptibility increase by KO mutation (AUC=0.76)	Tobramycin susceptibility decrease by KO mutation (AUC=0.77)
PA0334	PA0140	PA0011	PA0479	PA0392	PA0023
PA0336	PA0287	PA0401	PA0807	PA4077	PA0028
PA0337	PA0355	PA0402	PA0908	PA5199	PA0071
PA0338	PA0386	PA0420	PA0958	PA5366	PA0072
PA0425	PA0613	PA0427	PA1553	PA3649	PA0113
PA0426	PA0615	PA0503	PA3667	PA2656	PA0114
PA0427	PA0616	PA0764		PA5200	PA0149
PA0702	PA0617	PA0766		PA0016	PA0150
PA0703	PA0618	PA1195		PA4941	PA0151
PA0966	PA0619	PA2128		PA4315	PA0227
PA1098	PA0620	PA2615		PA2737	PA0305
PA1375	PA0621	PA2963		PA4942	PA0306
PA1588	PA0622	PA3050		PA0011	PA0307
PA1611	PA0623	PA3262		PA5054	PA0337
PA1667	PA0624	PA3433		PA5053	PA0338
PA1777	PA0626	PA3649		PA3014	PA0382
PA1800	PA0630	PA3800		PA4398	PA0432
PA1801	PA0633	PA3818		PA5528	PA1320
PA1802	PA0634	PA3978		PA2658	PA1321
PA1803	PA0636	PA4005		PA3735	PA1479
PA2432	PA0638	PA4007		PA5429	PA1480
PA2549	PA0640	PA4069		PA5130	PA1483
PA2615	PA0641	PA4088		PA3210	PA1547
PA3516	PA0647	PA4393		PA1805	PA1548
PA3517	PA0736	PA4745		PA3657	PA1549
PA3738	PA0737	PA5130		PA0762	PA1550
PA4459	PA0926	PA5174		PA3016	PA1551
PA4667	PA0951	PA5288		PA5133	PA1552
PA4685	PA0952	PA5366		PA5344	PA1553
PA4781	PA1259			PA0667	PA1554
PA5253	PA1345			PA4223	PA1555
PA5280	PA1428			PA1800	PA1556
PA5345	PA1433			PA5428	PA1588
PA5366	PA1434			PA4387	PA1589
PA5375	PA1513			PA4960	PA1621

PA1634
PA1987
PA2047
PA2060
PA2399
PA2400
PA2490
PA2566
PA2567
PA2638
PA2639
PA2642
PA2643
PA2644
PA2645
PA2647
PA2649
PA2682
PA2864
PA3003
PA3214
PA3276
PA3574
PA3620
PA3784
PA3835
PA3836
PA3958
PA4222
PA4223
PA4225
PA4400
PA4600
PA4658
PA4687
PA4688
PA4734
PA4946
PA5028

PA0374	PA1622
PA3013	PA1688
PA5471	PA1689
PA0502	PA1734
PA3194	PA1766
PA3485	PA1767
PA1775	PA1768
PA5261	PA1856
PA0966	PA1882
PA5256	PA1883
PA0014	PA2638
PA4404	PA2639
PA0592	PA2640
PA0375	PA2641
PA3050	PA2642
PA5368	PA2643
PA0427	PA2644
PA4222	PA2645
PA2812	PA2647
PA4426	PA2648
PA0662	PA2649
PA5493	PA2960
PA3824	PA2963
PA5369	PA2992
PA3823	PA2993
PA0411	PA2994
PA4422	PA2995
PA4410	PA2996
PA5351	PA2997
PA5227	PA2999
PA2018	PA3002
PA5285	PA3003
PA2604	PA3114
PA5367	PA3115
PA5175	PA3231
PA0413	PA3232
PA5194	PA3233
PA5551	PA3274
PA0664	PA3392

PA5131
PA5250
PA5334
PA5427
PA5551
PA5560
PA5562
PA5565

PA5241	PA3393
PA0298	PA3394
PA5479	PA3395
PA5475	PA3405
PA0424	PA3406
PA2019	PA3407
PA0663	PA3408
PA0047	PA3492
PA5406	PA3493
PA4050	PA3495
PA0108	PA3589
	PA3620
	PA3667
	PA3814
	PA3816
	PA3817
	PA3818
	PA3975
	PA3976
	PA4112
	PA4234
	PA4429
	PA4430
	PA4431
	PA4496
	PA4543
	PA4544
	PA4571
	PA4609
	PA4640
	PA4673
	PA4695
	PA4696
	PA4833
	PA4863
	PA4868
	PA4882
	PA4883
	PA4916

PA4917
PA4946
PA5001
PA5066
PA5067
PA5070
PA5145
PA5147
PA5300
PA5309
PA5312
PA5315
PA5334
PA5349
PA5350
PA5438
PA5447
PA5448
PA5450
PA5451
PA5452
PA5530

Supplementary Table S7. GEO expression data sets incorporated into PseudomonasNet

GEO series	Title	# Samples	# Nodes	# Links
GSE12207	Biofilms and type III secretion are not mutually exclusive in Pseudomonas aeruginosa[25]	15	980	9,448
GSE21966	Transcriptional profiling of P. aeruginosa isolated from 3 individuals with cystic fibrosis over time[26]	38	2,987	32,508
GSE25481	Analysis of Pseudomonas aeruginosa evolving in the cystic fibrosis lung uncovers limited evidence that mutator lineages are more genetically variable than non-mutator lineages[27]	10	549	10,475
GSE31227	Expression data of Pseudomonas aeruginosa isolated from cystic fibrosis patients in Denmark[28]	78	1,466	12,483
GSE33188	Expression data from Pseudomonas aeruginosa PAO1 and its isogenic ampR mutant in the presence and absence of sub-MIC beta-lactam exposure[29]	123	1,045	2,477
GSE33245	Novel targets of the CbrAB/Crc carbon catabolite control system revealed by transcript abundance in Pseudomonas aeruginosa	16	1,571	5,466
GSE4026	A distinct QscR regulon in the Pseudomonas aeruginosa quorum sensing circuit[30]	30	1,074	4,485
GSE6741	Response of Pseudomonas aeruginosa to low oxygen [31]	8	1,017	5,478
GSE6769	Expression data from Pseudomonas aeruginosa (wild type and lasRrhlR mutant strains) exposed to human neutrophils[32]	10	1,287	11,461
GSE8953	Investigations of the effects of subMIC antibiotics on the P. aeruginosa transcriptome[33]	12	956	12,512
GSE7704	In vivo evidence of Pseudomonas aeruginosa nutrient acquisition and pathogenesis in the cystic fibrosis lung[34]	14	2,485	10,480
GSE28081	Expression pattern of Pseudomonas aeruginosa biofilm-residing bacteria exposed to gentamicin or surface acoustic waves or gentamicin and surface acoustic waves combined	8	1,835	22,513

Supplementary References

1. Lee, I., Date, S.V., Adai, A.T. & Marcotte, E.M. A probabilistic functional network of yeast genes. *Science* **306**, 1555-8 (2004).
2. Lee, I. *et al.* A single gene network accurately predicts phenotypic effects of gene perturbation in Caenorhabditis elegans. *Nat Genet* **40**, 181-8 (2008).
3. Stapley, B.J. & Benoit, G. Biobibliometrics: information retrieval and visualization from co-occurrences of gene names in Medline abstracts. *Pac Symp Biocomput*, 529-40 (2000).
4. Barrett, T. *et al.* NCBI GEO: archive for functional genomics data sets--update. *Nucleic Acids Res* **41**, D991-5 (2013).
5. Mitchell, A. *et al.* The InterPro protein families database: the classification resource after 15 years. *Nucleic Acids Res* **43**, D213-21 (2015).
6. Date, S.V. & Marcotte, E.M. Discovery of uncharacterized cellular systems by genome-wide analysis of functional linkages. *Nat Biotechnol* **21**, 1055-62 (2003).
7. Shin, J., Lee, T., Kim, H. & Lee, I. Complementarity between distance- and probability-based methods of gene neighbourhood identification for pathway reconstruction. *Mol Biosyst* **10**, 24-9 (2014).
8. Kim, E., Kim, H. & Lee, I. JiffyNet: a web-based instant protein network modeler for newly sequenced species. *Nucleic Acids Res* **41**, W192-7 (2013).
9. Sonnhammer, E.L. & Ostlund, G. InParanoid 8: orthology analysis between 273 proteomes, mostly eukaryotic. *Nucleic Acids Res* **43**, D234-9 (2015).
10. Kim, H., Shim, J.E., Shin, J. & Lee, I. EcoliNet: a database of cofunctional gene network for Escherichia coli. *Database (Oxford)* **2015**(2015).
11. Willis, R.C. & Hogue, C.W. Searching, viewing, and visualizing data in the Biomolecular Interaction Network Database (BIND). *Curr Protoc Bioinformatics* **Chapter 8**, Unit 8 9 (2006).
12. Salwinski, L. *et al.* The Database of Interacting Proteins: 2004 update. *Nucleic Acids Res* **32**, D449-51 (2004).
13. Orchard, S. *et al.* The MIntAct project--IntAct as a common curation platform for 11 molecular interaction databases. *Nucleic Acids Res* **42**, D358-63 (2014).
14. Goll, J. *et al.* MPIDB: the microbial protein interaction database. *Bioinformatics* **24**, 1743-4 (2008).
15. Rain, J.C. *et al.* The protein-protein interaction map of Helicobacter pylori. *Nature* **409**, 211-5 (2001).
16. Titz, B. *et al.* The binary protein interactome of Treponema pallidum--the syphilis spirochete. *PLoS One* **3**, e2292 (2008).
17. Sato, S. *et al.* A large-scale protein protein interaction analysis in Synechocystis sp. PCC6803. *DNA Res* **14**, 207-16 (2007).
18. Shimoda, Y. *et al.* A large scale analysis of protein-protein interactions in the nitrogen-fixing bacterium Mesorhizobium loti. *DNA Res* **15**, 13-23 (2008).

19. Parrish, J.R. *et al.* A proteome-wide protein interaction map for Campylobacter jejuni. *Genome Biol* **8**, R130 (2007).

20. Rajagopala, S.V. *et al.* The binary protein-protein interaction landscape of Escherichia coli. *Nat Biotechnol* **32**, 285-90 (2014).

21. Arifuzzaman, M. *et al.* Large-scale identification of protein-protein interaction of Escherichia coli K-12. *Genome Res* **16**, 686-91 (2006).

22. Butland, G. *et al.* Interaction network containing conserved and essential protein complexes in Escherichia coli. *Nature* **433**, 531-7 (2005).

23. Hu, P. *et al.* Global functional atlas of Escherichia coli encompassing previously uncharacterized proteins. *PLoS Biol* **7**, e96 (2009).

24. Kuhner, S. *et al.* Proteome organization in a genome-reduced bacterium. *Science* **326**, 1235-40 (2009).

25. Mikkelsen, H. *et al.* Biofilms and type III secretion are not mutually exclusive in Pseudomonas aeruginosa. *Microbiology* **155**, 687-98 (2009).

26. Huse, H.K. *et al.* Parallel evolution in Pseudomonas aeruginosa over 39,000 generations in vivo. *MBio* **1**(2010).

27. Warren, A.E. *et al.* Genotypic and phenotypic variation in Pseudomonas aeruginosa reveals signatures of secondary infection and mutator activity in certain cystic fibrosis patients with chronic lung infections. *Infect Immun* **79**, 4802-18 (2011).

28. Yang, L. *et al.* Bacterial adaptation during chronic infection revealed by independent component analysis of transcriptomic data. *BMC Microbiol* **11**, 184 (2011).

29. Balasubramanian, D. *et al.* The regulatory repertoire of Pseudomonas aeruginosa AmpC ss-lactamase regulator AmpR includes virulence genes. *PLoS One* **7**, e34067 (2012).

30. Lequette, Y., Lee, J.H., Ledgham, F., Lazdunski, A. & Greenberg, E.P. A distinct QscR regulon in the Pseudomonas aeruginosa quorum-sensing circuit. *J Bacteriol* **188**, 3365-70 (2006).

31. Alvarez-Ortega, C. & Harwood, C.S. Responses of Pseudomonas aeruginosa to low oxygen indicate that growth in the cystic fibrosis lung is by aerobic respiration. *Mol Microbiol* **65**, 153-65 (2007).

32. Alhede, M. *et al.* Pseudomonas aeruginosa recognizes and responds aggressively to the presence of polymorphonuclear leukocytes. *Microbiology* **155**, 3500-8 (2009).

33. Skindersoe, M.E. *et al.* Effects of antibiotics on quorum sensing in Pseudomonas aeruginosa. *Antimicrob Agents Chemother* **52**, 3648-63 (2008).

34. Son, M.S., Matthews, W.J., Jr., Kang, Y., Nguyen, D.T. & Hoang, T.T. In vivo evidence of Pseudomonas aeruginosa nutrient acquisition and pathogenesis in the lungs of cystic fibrosis patients. *Infect Immun* **75**, 5313-24 (2007).
